# Childhood abuse and maladaptive coping in care leavers: An exploratory study on attachment and Early Maladaptive Schemas

**DOI:** 10.1177/13674935251341921

**Published:** 2025-05-26

**Authors:** Melanie Jarvis, Katy Sivyer, Emma Selwood, Kate Willoughby

**Affiliations:** 1 University of Southampton - Highfield Campus, Southampton, United Kingdom of Great Britain and Northern Ireland; 2 15577 Southampton City Council, Southampton, United Kingdom of Great Britain and Northern Ireland

**Keywords:** Attachment, care leavers, child abuse, early maladaptive schemas, maladaptive coping

## Abstract

Care leavers face adversity and poor psychological outcomes, despite being a significantly under researched population. Childhood maltreatment is associated with attachment insecurity and Early Maladaptive Schemas (EMS); however, there is little research into the relationships between these variables, and none exploring these variables in care leavers. The study aimed to investigate the relationship between childhood maltreatment, attachment, EMS and maladaptive coping in care leavers, hypothesising that higher levels of childhood maltreatment would be associated with increased attachment insecurity, EMS severity and maladaptive coping. Participants were 53 UK-based adults, with experience of living in care during childhood. An opportunity sample and a within-subjects, cross-sectional survey design was used with hierarchical multiple regression. High levels of childhood maltreatment, attachment insecurity (both anxious and avoidant), EMS and maladaptive coping were found within the sample of care leavers. Hierarchical regression indicated that both attachment anxiety and attachment avoidance, and EMS domain ‘disconnection and rejection’ were significant predictors in the relationship between childhood maltreatment and maladaptive coping. The study adds to the current knowledge about psychological vulnerabilities for care leavers. Results highlight the importance of targeted assessment, formulation and psychological interventions targeting attachment domains and EMS, with particular focus on the disconnection and rejection schema domain for this population.

## Introduction

### Care leavers

Epidemiological studies have revealed that care leavers (defined as anybody who has spent time in foster care during childhood) in the UK fare worse than their peers across a variety of domains including educational attainment (Fletcher et al., 2015; O’Higgins et al., 2015) and mental health (Beagley et al., 2014). Care leavers are widely recognised as having significant vulnerabilities and often do not receive necessary support (Bromley et al., 2020; Care Quality Commission, 2016; McAuley and Davis, 2009). High rates of childhood abuse have been reported within care leavers (Bazalgette and Trevelyan, 2015; Department for Education, 2014), and such abuse experiences have been associated with a range of psychological difficulties (Ball and Links, 2009; McFetridge et al., 2015).

Care leavers are a significantly under researched and hard to reach population (Smith, 2017). Previous research has focussed on outcomes for care leavers (e.g. educational attainment, housing attainment and spending time in custody (Harrison et al., 2020), with little research on why vulnerabilities exist within this population (Dixon, 2008). There are no studies that have explored childhood maltreatment, attachment and EMS variables within this population. The present study aimed to explore the relationship between childhood maltreatment, attachment, EMS and maladaptive coping.

### Attachment theory

Webster and Hackett (2007) have argued that attachment theory can provide a useful framework for conceptualising difficulties relating to care leavers. Attachment theory highlights that a child’s early experience with a primary caregiver creates the framework for future interpersonal relationships and emotional regulation abilities (Bowlby, 1973, 1984). According to Bowlby (1973), children construct mental representations known as ‘Internal Working Models’ (IWMs), which allow their thoughts and feelings to be organised at times when the attachment system is put under stress (e.g. when a child is separated from a caregiver). When parenting has been sensitive, positive expectations about others’ availability and positive views of the self are formed and attachment security is achieved (Mikulincer et al. 2003).

For those who have experienced abuse, attachment systems may develop differently, for example, if caregivers have been violent, insensitive or unavailable, IWMs will be constructed accordingly, and defensive processes will be developed to help protect from painful thoughts and feelings (Bowlby, 1984). This creates an insecure attachment. An insecure attachment refers to a category of attachment style characterised by a fear of intimacy, fear of abandonment, lack of trust and lack of a secure base (Mikulincer et al. (2003). Historically, three primary types of insecure attachment styles have been categorised: avoidant attachment, anxious attachment and disorganised attachment; however, the literature on attachment stability over time has highlighted some inconsistencies, from moderate stability (Fraley, 2002) to no stability across larger time intervals (Pinquart et al., 2013). As a result, Fraley et al. (2000b) favoured measuring attachment as a dimension rather than a discrete category (i.e. measuring level of attachment anxiety or level of attachment avoidance). The present study will use these dimensions to measure attachment.

### Childhood abuse, attachment and psychological difficulties

Empirical and theoretical links between childhood abuse and attachment insecurity have been well established (Aspelmeier et al., 2007; Banyard et al., 2017; Winham et al., 2015). Attachment insecurity has been associated with several psychological and behavioural difficulties including maladaptive coping (Perlman et al., 2016; Shorey and Snyder, 2006), emotional dysregulation (Mikulincer and Shaver, 2007) and personality disorder (Herman et al., 1989). Although a causal direction is unclear, correlations between childhood abuse experience, attachment insecurity and psychological difficulties in adulthood have been observed (Aspelmeier et al., 2007; Perlman et al., 2016; Winham et al., 2015).

For care leavers, risk of attachment insecurity and associated psychological distress is significant (McAuley and Davis, 2009). Care leavers are likely to have experienced abuse prior to entering care (Bazalgette and Trevelyan, 2015) in addition to experiencing loss of attachment and placement instability once in care (Hannon et al., 2010). These factors in turn can serve to reinforce the attachment difficulties previously encountered and perpetuate the psychological difficulties that follow (Hannon et al., 2010; Ward et al., 2010).

### Schema theory and early maladaptive schemas

Similar to IWMs, EMS are defined as a broad pervasive pattern relating to oneself and one’s relationships, developed during childhood and rehearsed throughout the lifetime (Young et al., 2003). They are made up of memories, bodily sensations, emotions and cognitions, which once activated evoke intense emotional responses. Young et al. (2003) proposed five categories of schema domains from 18 EMS (see Supplemental materials for a table summary). When activated, EMS are thought to drive behaviour and coping in a pattern which then further perpetuate the schema (Young, 1994).

Several authors have observed relationships between IWMs and schemas (Bosmans et al., 2010; Mason et al., 2005). It is thought that attachment theory offers an explanation for how early experiences shape internal working models about self and others, whereas schema therapy provides a framework for classifying challenging beliefs and coping behaviours that may arise from those early experiences. Where attachment theory focusses on pre-verbal and unconscious relational processes, EMS encompasses conscious cognitive processes.

### Childhood abuse and Early Maladaptive Schemas

Emerging evidence suggests EMS are correlated with childhood experiences of abuse (Lumley and Harkness, 2007; Pilkington et al., 2021; Wright et al., 2009). Following abuse experiences, a prominence of the ‘disconnection and rejection’ schema domain has been observed, over and above other schema domains (Cecero et al., 2004; Gay et al., 2013; McGinn et al., 2005). This schema domain has been associated with the highest levels of psychological distress (Furnivall et al., 2012; Murphy, 2011).

### Childhood abuse and coping

Research has found individuals who have experienced childhood abuse use more maladaptive coping strategies including avoidant coping (Walsh et al., 2010), disengagement (Dishoom-Brown et al., 2017; Leitenberg et al., 2004), denial and self-blame (Dishon-Brown et al., 2017) and substance use (Dishon-Brown et al., 2017; Filipas and Ullman, 2006; Logan et al., 2006). Attachment insecurity has been associated with both a lack of adaptive coping strategies (Kobak and Sceery, 1988; Mikulincer et al., 2003) and increased maladaptive coping (Marganska et al., 2013; Mikulincer et al. 2003). Similarly, EMS are thought to drive behaviour and maladaptive coping following abuse experiences (Young et al., 2003).

### Aim

The aim was to evaluate the relationship between childhood maltreatment, attachment, EMS and maladaptive coping in care leavers.

## Method

The study employed a cross-sectional survey design using online and paper-based questionnaires.

### Recruitment

A self-selecting sample was used to recruit participants. Inclusion criteria were adults who had some experience of living in foster care as a child. Exclusion criteria were children or those without experience of foster care. Recruitment took place via third sector organisations, charities and online social media platforms (Instagram, Twitter and Facebook). Willing participants self-selected by emailing the researcher. Participants were offered a £5 Amazon voucher upon completion of the questionnaires.

### Measures

#### Childhood maltreatment

The Child Abuse and Trauma Scale (CATS; Sanders and Becker-Lausen, 1995) was used to assess childhood abuse. The CATS is a 38-item self-report questionnaire used to identify the frequency and severity of different types of childhood maltreatment (negative home environment/neglect, emotional abuse, physical abuse and sexual abuse). The measure has been found to have satisfactory psychometric properties with test–retest reliability (r = 0.71–0.91), concurrent validity (r = 0.24–0.41) and internal consistency (α = 0.63−0.88; Kent and Waller, 1998; Sanders and Becker-Lausen, 1995).

#### Attachment anxiety and avoidance

Attachment anxiety and avoidance was assessed using the Experience in Close Relationships-Relationship Structures Questionnaire (ECR-RS: Fraley et al., 2006). The ECR-RS is a 36-item self-report questionnaire derived from the Experience in Close Relationships-Revised (ECR-R; Fraley et al., 2000b). It measures attachment patterns across general and specific relationships (mother, father, romantic partner and best friend) using nine items to assess anxious and avoidant dimensions of attachment. Global attachment avoidance and attachment anxiety can be established by calculating the average of the relevant scores for four individual targets. Internal consistency for both the anxiety and the avoidance scales has been shown to be greater than or equal to 0.89 (Fraley et al., 2006).

#### Early Maladaptive Schemas

EMS were assessed using the Young Schema Questionnaire – Short Form 3 (YSQ-SF3; Young, 2005). The measure is a 90-item self-report questionnaire measuring 18 different EMS across the five schema domains. The YSQ, in both its long and short forms, has proven to hold good psychometric properties (Lee et al., 1999). For the YSQ-SF3, Cronbach’s alpha level was 0.96 in a clinical sample (Waller et al., 2001).

#### Coping

Coping was assessed through the COPE Inventory (Carver et al., 1989), a 60-item self-report questionnaire which comprises 14 discrete coping subscales based on theoretical categories of coping. Maladaptive and adaptive coping categories have been previously derived from the COPE (Meyer, 2001; Perlman et al., 2016), with Cronbach’s alpha values of greater than 0.7 with these categories (Perlman et al., 2016). The COPE has been found to have good internal reliability (Shapiro and Levendosky, 1999).

### Statistical analysis strategy

Data were analysed using IBM SPSS Statistics (Version 24). A hierarchical multiple regression was used to examine the relationship between childhood maltreatment, attachment (anxiety and avoidance), EMS and maladaptive coping. For the regression analyses, multiple imputation, using five imputed datasets, was used to assess the impact of missing data (Feng et al. 2021).

## Results

### Analyses

A total sample of 53 was achieved; however, only 39 of the total 53 participants (75%) returned completed YSQ-SF3 questionnaires, lowering the data for EMS variables. There were no significant differences between completers and non-completers (Little’s MCAR test (*p* = 0.962)). Data were broadly normally distributed, although there were some outliers, but the decision was made to retain them as they were not consistently outlying across the measures and were considered severe cases within this population.

### Descriptive statistics

Demographic characteristics are displayed in Table 1 and descriptive statistics in Supplemental materials. High levels of child maltreatment, attachment insecurity, EMS and maladaptive coping were found in the sample. A high total CAT score was found (M = 2.32, SD = 0.79) which is more than double of that found in various non-clinical samples (M = 0.39 to 0.91, SD = 0.06 to 0.66; Kent and Waller, 1998; Sanders and Becker-Lausen, 1995). Both attachment anxiety and avoidance scores are significantly above those found within non-clinical populations (M = 3.18, SD = 0.96 and M = 2.53, SD = 1.19, respectively; Fraley et al., 2011) and clinical populations (M = 3.63, SD = 1.80 and M = 3.19, SD = 1.43, respectively; Selwood, 2013). Mean scores for total EMS score (M = 3.20, SD = 2.70) were higher than that found in a sample categorised with secure attachment (M = 2.52, SD = 0.86, Mason et al., 2005). The disconnection and rejection domain was significantly higher than that found within a non-clinical sample (M = 2.14, SD = 0.84; Mairet et al., 2014). Higher levels of both adaptive and maladaptive coping were found compared with non-clinical samples (adaptive; M = 1.65; and maladaptive; M = 0.95; Moore et al., 2011).

**Table 1.** Demographic characteristics of sample.

Variable	Sub-category	M/IQR	*n* (%)
Age (years)	18–35	Median = 33	33 (61.1%)
	36–55	IQR = 16	17 (31.5%)
	56+		3 (5.6%)
Gender	Male		22 (41.5%)
	Female		31 (58.5%)
Ethnicity	White British		28 (52.8)
	White other		8 (15.1%)
	Black African		5 (9.4%)
	Black Caribbean		2 (3.8%)
	Other Black		3 (5.7%)
	Indian		1 (1.9%)
	Pakistani		1 (1.9%)
	Chinese		1 (1.9%)
	Other Asian		2 (3.8%)
	Did not state		1 (1.9%)
Age entered care	From birth to 3 years		16 (30.3%)
	4–8 years		9 (16%)
	9–14 years		20 (37.7%)
	15–18 years		8 (15.1%)
Time spent in care	From birth to 2 years		25 (26%)
	3–6 years		16 (30%)
	7–10 years		9 (17 %)
	11–14 years		7 (12%)
	15–18 years		7 (13.2%)
Number of placements	1–3		28 (52.9%)
	4–8		16 (30.2%)
	9+		9 (17%)

^a^*N* = 53.

### Hierarchical regression analyses

The hierarchical multiple regression revealed that childhood maltreatment alone (model 1) did not contribute to a significant regression model, (F (1, 51) = 3.06, *p* = 0.86) and accounted for 6% of the variation in maladaptive coping. Introducing attachment variables (model 2) explained an additional 18% in variation of maladaptive coping, and this change in R^2^ was significant (*F* (2,49) = 5.82, *p* = 0.005). Adding schema domains to the regression (model 3) explained an additional 23% and this change to R^2^ was also significant (*F* (5,44) = 3.82, *p* < 0.006). When all eight variables were included in stage 3 of the regression model, childhood maltreatment and all the schema domains, except for the disconnection and rejection schema domain, were not significant predictors of maladaptive coping. Together the variables accounted for 47% of the variance of maladaptive coping.

Regression statistics are shown in Table 2.

**Table 2.** Multiple hierarchical regression coefficients predicting maladaptive coping.^a^

		Unstandardised coefficients					95% confidence interval for B	
		B	Std. E	Standardised coefficients beta	t	Sig	Lower bound	Upper bound
Step 1	(Constant)	56.03	4.56		12.28	<.001	46.86	65.19
	Childhood maltreatment	0.09	0.05	0.24	1.75	0.09	−0.01	0.19
Step 2	(Constant)	60.27	5.83		10.34	<0.001	48.56	71.98
	Childhood maltreatment	0.12	0.06	0.33	2.14	0.037	0.01	0.23
	Attachment avoidance	−0.29	0.09	−0.53	−2.64	0.011	−0.40	−0.054
	Attachment anxiety	0.36	0.11	0.58	3.35	0.002	0.14	0.57
Step 3	(Constant)	49.99	7.60		6.58	<0.001	34.67	65.31
	Childhood maltreatment	0.11	0.05	0.30	2.03	0.048	0.01	0.26
	Attachment avoidance	−0.27	0.08	−0.62	−3.42	0.001	−0.43	−0.11
	Attachment anxiety	0.33	0.10	0.53	3.32	0.002	0.13	0.52
	Impaired autonomy	−0.10	0.12	−0.15	−.81	0.42	−0.35	0.15
	Impaired limits	−0.30	0.17	−0.23	−1.79	0.08	−0.64	0.04
	Over vigilance	−0.03	0.11	−0.05	−.26	0.80	−0.25	0.19
	Other directedness	0.31	0.17	0.32	1.79	0.08	−0.04	0.66
	Disconnection and rejection	0.20	0.07	0.49	2.77	0.008	0.06	0.35

^a^Five multiple imputations were used showing broadly similar results. Table presents data from first imputation model; B = unstandardised coefficient; Std E = standard error of B (unstandardised coefficient). Total *N* = 53; for schema data *n* = 39.

## Discussion

The study aimed to investigate the relationship between childhood maltreatment, attachment, EMS and maladaptive coping in care leavers. Notably, there was a small sample size within the study, meaning that results are likely to have been underpowered. Despite this, results imply some possible early findings and trends, which would benefit from further research. High levels of childhood maltreatment, attachment insecurity (both anxious and avoidant), EMS and maladaptive coping were found within the sample of care leavers. 17% of participants experienced more than nine foster care placements, highlighting a significant vulnerability.

Results from the hierarchical regression analyses indicated that childhood maltreatment alone did not predict maladaptive coping. However, when attachment anxiety and avoidance together were added, the model became significant in predicting the relationship between childhood maltreatment and maladaptive coping. When all five categories of EMS were included within the model alongside attachment anxiety and avoidance, the overall model remained significant in predicting the relationship between childhood maltreatment and maladaptive coping; however as individual variables, only attachment (anxiety and avoidance) and the disconnection and rejection schema domain significantly predicted the relationship between childhood maltreatment and maladaptive coping.

Both attachment anxiety and avoidance were significant predictors of maladaptive coping; however, these were in the opposite direction. Attachment anxiety significantly positively related to higher levels of maladaptive coping, whereas attachment avoidance significantly negatively related to maladaptive coping, suggesting that attachment avoidance decreased maladaptive coping. Again, this finding may be a result of the study being underpowered; however, a further possible interpretation for this result relates to the differences in strategies employed by anxious versus avoidant individuals, given the evidence that attachment avoidance symptomology is less pronounced than attachment anxiety (Declercq and Willemsen, 2006; Mason et al., 2005). A significant positive relationship between maladaptive coping and attachment anxiety is consistent with previous literature among individuals with childhood abuse experiences (Perlman et al., 2016). Attachment anxiety is associated with negative self-evaluations, preoccupation with attachment related goals and impulsivity (Mikulincer and Shaver, 2007). As a result, individuals with attachment anxiety are more likely to use more self-blame and have higher levels of guilt and shame following the experience of abuse (Murthi et al., 2006), leading to development of maladaptive emotion regulation strategies and ineffective coping at times of attachment activation (Mikulincer and Shaver, 2007). Attachment avoidance, on the other hand, is associated with inhibition of thoughts or emotions (Mikulincer and Shaver, 2007). There is evidence that attachment avoidance symptomology can present as less pronounced than attachment anxiety (Declercq and Willemsen, 2006; Mason et al., 2005) and that participants with attachment avoidance tend to under-report psychological symptoms when compared to reports given by people who know them well (Dozier and Lee, 1995). The ECR-RS may not have been a sensitive enough measure for attachment avoidance within this context (see limitations).

The disconnection and rejection schema domain was the only EMS domain that significantly predicted maladaptive coping within this study, which may be a result of analyses being underpowered. However, the prominence of the disconnection and rejection domain is consistent with data from survivors of childhood abuse (Cecero et al., 2004; McGinn et al., 2005) and within care leaver populations specifically (Murphy, 2011), and it fits previous findings which have isolated this domain as a unique variable in the relationship between childhood abuse and psychological difficulties (Bosmans et al., 2010; Gay et al., 2013; Murphy, 2011). It has been suggested that the disconnection and rejection schema domain, in particular, is associated with childhood abuse and those with high prominence within this domain are the most impaired (Young et al., 2003), highlighting possible specific vulnerabilities within the care leaver population.

## Limitations

The study is one of few studies examining the psychological needs of care leavers and was the first to examine the relationship between childhood abuse, attachment and EMS and maladaptive coping within this population. The cross-sectional design of the study means that it is not possible to infer causality; however, the results and limitations help pave the way for future research directions.

Most significantly, the study was limited by a small sample size, meaning analyses were likely underpowered, and therefore some of the relationships may have been erroneously ruled out. Although this is a significant limitation, it is important to note that, given the difficulties in researching this population, the study acts as an important starting place for necessary future research.

Another limitation was that the study used a self-selecting sampling design, meaning it may have been more appealing to care leavers based on a range of factors including their attachment orientation and level of adaptive coping. Future research would benefit from enhanced and robust recruitment approaches that target services that have existing relationships with care leavers (such as social care teams), particularly for care leavers who present with attachment and relational needs.

There was no matched non-care leaver control group, meaning it is not possible to ascertain that findings are entirely exclusive to care leaver populations. Reliance on a self-report measure of attachment may have also impacted on results, specifically relating to attachment avoidance. Given that people with avoidant attachment tend to minimise the impact of historical experiences, self-report questionnaires that rely on conscious processes may not have been appropriately sensitive to pick up effects within this group (Mikulincer and Shaver, 2007).

## Implications for practice

Despite the small sample within this study, results indicate some possible vulnerabilities for care-leavers, including frequent placement change, high levels of attachment insecurity, EMS and maladaptive coping, all of which indicate a possible need for psychological interventions that target these areas. Examples of these include Schema-Focused Therapy (Young, 1994) and Cognitive Analytic Therapy (Ryle, 1979), which focus on understanding one’s internal processes and early belief system development to change current patterns of maladaptive coping.

Findings indicate that it may be helpful for clinicians supporting care leavers to assess individual attachment style and EMS prior to formulating a treatment plan. With regards to psychological support, attachment insecurity has been associated with poor engagement (Muller et al., 2008) and poor outcomes (Stalker et al., 2005), which is one likely explanation for the difficulties that care leavers face engaging with mental health services (Lamont et al., 2009). Therapeutic interventions for this population likely need to provide more flexible approaches, with an additional focus on the role of interpersonal relationships. Therapeutic approaches tailored to the needs and strategies associated with each attachment style are likely to be beneficial. For example, for those presenting with high levels of attachment anxiety, support with emotional regulation and co-regulation with the therapist will be essential. Whereas those presenting with high levels of attachment avoidance may need more assertive outreach and will need to be gently supported to build affective expression and interpersonal connectedness (Tasca et al., 2009). Therapeutic approaches may be complimented by community level and peer support (i.e. support groups, youth clubs and mentoring schemes), which support care leavers to develop their interpersonal and attachment relationships more broadly.

Given that individuals with insecure attachments will have difficulties trusting others (Dugal et al., 2016), a focus on relational factors such as warmth, validation and consistency will be central. Attachment-based, relational or systemic approaches may also be favoured over behavioural or cognitive approaches (Becker-Weidman and Hughes, 2008). Indeed, emerging literature has highlighted that attachment insecurity can be changed over time (Saunders et al., 2011) and changes in attachment scores have been found within therapeutic relationships (Smith et al., 2010) and following interventions (Becker-Weidman and Hughes, 2008; Elklit, 2009).

Findings support the exploration of EMS or use of schema therapy (Young et al., 2003) as a supportive therapeutic intervention for care leavers. Young et al. (2003) suggested that individuals with prominent EMS within the disconnection and rejection domain will likely struggle to form therapeutic relationships easily due to fears of rejection. As such, care leavers are likely to have difficulties engaging and may not be adequately served in services where strict policies of attendance are enforced (Murphy, 2011). In practice, it may be beneficial for practitioners to complete early assessment of EMS to support treatment planning that is flexible and relationally focussed. For example, individuals that score highly for the disconnection and rejection domain are going to need a slow and sensitive approach to relationship building to work through some of these difficulties (Young, 2005) prior to working on behaviour change or developing new ways of coping. It is promising that there is emerging evidence that Schema Therapy reduces schema severity and associated symptomology within clinical populations (Cockram et al., 2010; Dickhaut and Arntz, 2014). Although there is currently no evidence base for the use of Schema Therapy for care leavers, it is likely to have something to offer this population.

## Future research

Replication using a larger and more representative sample, and an enhanced and robust approach to recruitment will be essential to generalise results. A larger sample may also allow for further investigation of specific categories of abuse and their relationship to attachment and EMS patterns. Future research may benefit from utilising more robust assessment tools of attachment, for example, the Adult Attachment Interview (Plotka, 2011). Longitudinal research is necessary to build a comprehensive understanding of how the domains explored develop over the lifespan for care leavers. This may also add insights that contribute to the timeliness of psychological provision. The use of an experimental design would be helpful to explore the efficacy of interventions targeting attachment style, the disconnection and rejection EMS domain and maladaptive coping in care leavers.

## Conclusion

Despite care leavers being recognised as some of the most vulnerable members of society (Care Quality Commission, 2016), there has been a lack of research investigating the psychological needs of this group (Dixon, 2008). The aim of the study was to investigate the relationship between childhood maltreatment, attachment, EMS and maladaptive coping in care leavers. The study identified high levels of childhood maltreatment, attachment insecurity (both anxious and avoidant), EMS and maladaptive coping within the sample of care leavers. Results indicated that both attachment anxiety and attachment avoidance, and the EMS domain ‘disconnection and rejection’ significantly predicted the relationship between childhood maltreatment and maladaptive coping. Childhood maltreatment alone did not predict maladaptive coping, neither did the remaining four EMS variables. The present study adds to the current knowledge about the relationship between early experiences, attachment style and later coping for care leavers, highlighting potential vulnerabilities in attachment insecurity and disconnection and rejection schema domain. The study had a small sample size and was likely underpowered. Further research is essential to validate and generalise the findings; however, results highlight importance of targeted assessment, formulation and psychological interventions targeting attachment domains and the disconnection and rejection schema domain.

## Supplemental Material

**Supplemental Material -** Childhood abuse and maladaptive coping in care leavers: An exploratory study on attachment and Early Maladaptive Schemas Supplemental Material for Childhood abuse and maladaptive coping in care leavers: An exploratory study on attachment and Early Maladaptive Schemas by Melanie Jarvis, Katy Sivyer, Emma Selwood and Kate Willoughby in Journal of Child Health Care.
