# Supplementary material for: Childhood abuse and maladaptive coping in care leavers: An exploratory study on attachment and Early Maladaptive Schemas
Source: J Child Health Care. 2025 May 26;30(2):334–48. doi: 10.1177/13674935251341921 (PMC13168622; doi:10.1177/13674935251341921)
Supplement: Supplemental Material - Childhood abuse and maladaptive coping in care leavers: An exploratory study on attachment and Early Maladaptive Schemas [file sj-pdf-1-chc-10.1177_13674935251341921.pdf]

Maladaptive Schema Domains (Based on Young et al. 2003)

Maladaptive Schema Domain	Home environment	Associated Beliefs and Behaviours
**Disconnection & Rejection**	Detached, unpredictable and abusive	Learn to expect that their need for safety, nurturance and empathy will not be met
**Impaired Autonomy and Performance**	Controlling or enmeshed	Learn not to trust their ability and so may struggle to function independently
**Impaired Limits**	Over indulgent or permissive	Deficiencies relating to internal limits, respect, and responsibility
**Other Directedness**	Conditional love and acceptance	Excessive motivation to meet the needs of others, at the expense of their own needs.
**Over Vigilance & Inhibition**	Dominant family structures or where performance standards and self-control took priority	Excessive focus of controlling, suppressing or ignoring their emotions in order to avoid making mistakes or to meet rigid internalised rules

Mood Repair Task

INSTRUCTIONS

This is an optional task which can be completed at any time after taking part in the research study.

Please read these general instructions completely before you begin writing.

Expressive writing can be a useful way to alleviate distress and promote emotional wellbeing. Pennebaker (2004) developed an expressive writing task to promote health and wellbeing in different groups of people, and his writing tasks have been replicated numerous of times with positive outcomes in relation to reducing distress, and promoting psychological wellbeing (Pennebaker, 1997; Krpan et al., 2013). Pennebaker (1997) highlights that the process of expressive writing can help people to give space to difficult feelings so that they can be processed, helping to ease emotional trauma.

The Pennbaker Paradigm (explained below) will guide you through an example of an expressive writing task. If you chose to complete the task your writing will not be shared with the researcher or anyone else. It is just for you.

Creative writing task

In your writing, I would like you to really let go and explore your very deepest emotions and thoughts about the difficult experiences in your life. You might tie this to other parts of your life: your childhood, your relationships with others, including parents, lovers, friends, relatives, or other people important to you. You might link your writing to your future and who you would like to become your future, or to who you have been, who you would like to be, or who you are now. Not everyone has had a single trauma, but all of us have had major conflicts or stressors, and you can write about these as well. All your writing is confidential. There will be no sharing of content. Do not worry about form or style, spelling, punctuation, sentence structure, or grammar.

1. **Time:** Write for approximately 20 minutes. You can repeat this as many times as you wish in the days to come.

2. **Topic**: What you choose to write about should be extremely personal and important to you.

3. **Write continuously:** Do not worry about punctuation, spelling, and grammar. If you run out of things to say, draw a line or repeat what you have already written. Keep pen on paper.

4. **Write only for yourself:** You may plan to destroy or hide what you are writing. Do not turn this exercise into a letter. This exercise is for your eyes only.

5. **Observe the Flip-out Rule**: If you get into the writing, and you feel that you cannot write about a certain event because it will push you over the edge, STOP writing!

6. Expect heavy boots: Many people briefly feel a bit saddened or down after expressive writing, especially on the first day or so. Usually this feeling goes away completely in an hour or two.

Give yourself sometime after writing to reflect on what you have written and to be compassionate with yourself. If you are worried about someone else seeing what you wrote, put your writing in a safe place, or simply tear it up or shred it. But if you are not concerned that someone may read what you wrote, you may want to keep your writing, so you can come back to it after you have completed.

Statistical Analyses Strategy

Data were analysed using IBM SPSS Statistics (Version 24). A total sample of 53 was achieved, however only 39 of the total 53 participants (75%) returned completed YSQ-SF3 questionnaires, lowering the data for EMS variables. There were no significant differences between completers and non-completers (Little's MCAR test (p=0.962). Normal distributions were shown across the majority of scales. Some outliers were identified using boxplots; however these were not removed as they were not consistently outlying across the measures and were considered severe cases within this population. Bootstrapping was used to manage this where appropriate.

Hierarchical regression analyses. A hierarchical multiple regression was used to examine the relationship between childhood maltreatment, attachment (anxiety and avoidance), EMS and maladaptive coping. For the regression analyses, multiple imputation, using five imputed datasets, were used to assess the impact of missing data (Feng, Hategeka & Grepin (2021).

A three-stage hierarchical multiple regression was conducted with maladaptive coping as the dependent variable. Childhood maltreatment was entered at stage one of the regression (model 1). Attachment avoidance and anxiety were then entered at stage two (model 2), given the established literature highlighting that attachment insecurity can arise out of childhood maltreatment experiences. Maladaptive schema domains were entered at stage three (model 3), given that EMS are cognitive processes they are likely to arise later out of earlier attachment representations.

Means and Standard Deviations for Measures of Childhood Maltreatment, Attachment and EMS.

	Chronbach's α	n	Range	Mean	Std. Deviation
Total maltreatment	**.94**	53	0-129	87.81	30.037
Neglect Total	.84	53	0-59	40.32	14.402
Physical Abuse	.80	53	0-22	17.42	6.347
Emotional Abuse	.84	53	0-28	16.43	7.148
Sexual Abuse	.71	53	0-21	8.42	5.729
Anxious attachment	**.87**	53	0-65	45.87	17.720
Avoidant attachment	**.88**	53	0-113	103.58	25.119
Total EMS	**97**	39	0-296	287.77	74.793
Disconnection & rejection	.95	39	0-101	86.44	28.691
Over vigilance	.92	39	0-93	71.90	20.513
Other directedness	.81	39	0-64	51.23	12.588
Impaired autonomy	.89	39	0-72	50.23	17.979
Impaired limits	.79	39	0-40	28.72	9.156
Total adaptive coping	.88	53	0-59	77.64	14.492
Total maladaptive coping	**.82**	53	0-47	63.58	10.871
